# Sombor topological indices for different nanostructures

**DOI:** 10.1016/j.heliyon.2023.e20600

**Published:** 2023-10-05

**Authors:** Muhammad Imran, Rashad Ismail, Muhammad Azeem, Muhammad Kamran Jamil, Esmail Hassan Abdullatif Al-Sabri

**Affiliations:** a Department of Mathematics, Riphah International University Lahore, Pakistan; b Department of Mathematics, Faculty of Science and Arts, King Khalid University, Muhayl Assir 61913, Saudi Arabia

**Keywords:** Sombor descriptors, Geometric indices, Nanostructures, Nano-junctions, Degree–based topological descriptors

## Abstract

Euclidean geometry is utilized to establish the Sombor graph parameter and its invariants. It is sum of all adjacent vertices in graph theory $\sqrt{d_{ϒ}^{2}+d_{\Gamma }^{2}}$ where $d_{ϒ}$ is the degree of the vertex ϒ. Geometrical interpretation is used to describe the new Sombor indices types. We examined, recently developed Sombor indices for various nanotube Y-junctions in this article. In specifically, the first area-based Sombor index was introduced by Euclidean geometry. Angular orientation concept to construct the second, fourth, and sixth Sombor graph parameters, while third and fifth Sombor graph parameters are constructed by perimeter.

## 1 Introduction

Topological indices also go by other names, including graph parameters based on vertex degree. Due to the transdisciplinary uses of topological indices, mathematicians and chemists are also drawn to this research issue. In the chemical database, nearly 3,000 topological indexes have been licensed since H. Wiener published the Wiener index in 1947 [1], [2].

**Figure 1:**
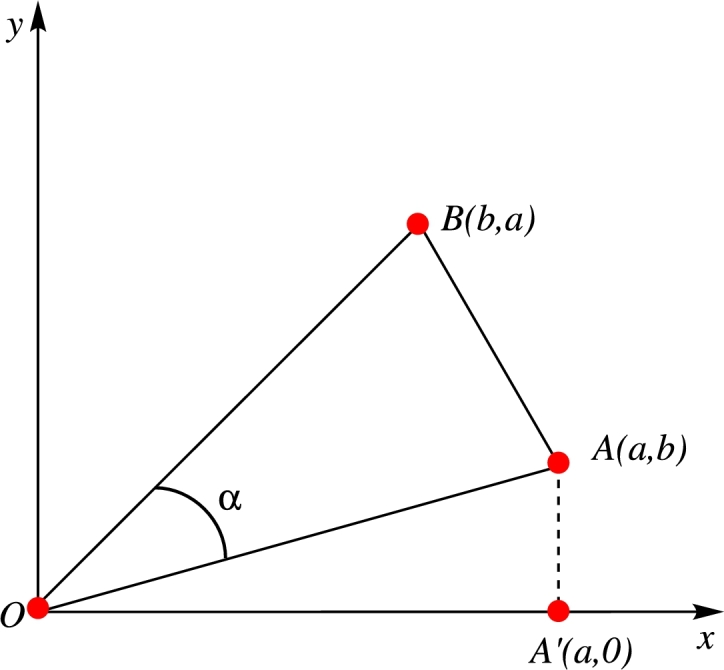
Geometrical Interpretation.

In the coordinate system for two dimensions (described in Fig. 1), the ordered pair (a,b) in ϒΓ∈E(G), is symbolized the edges degree points, and *a* is a degree of vertex ${ϒ}^{th}$, denoted by $d_{ϒ}$, similarly, *b* is the degree of the vertex $\Gamma^{th}$, represented by $d_{\Gamma }$. The coordinates (b,a) in Fig. 1 is the dual degree edge point. Assume that the geometric image *Q* has area is represented by area(Q), and perimeter is denoted by perim(Q), and let $B^{\prime }$ be the point of projection of point *Q* on the *x*-axis. For more detail see the citation of [3].

In [4], firstly presented the Sombor index, by applying the geometric interpretation. This index has been extensively studied because of its geometrical concerns [5], [6]. From a geometrical perspective, no other degree based vertex parameter has been are proposed.

Topological indices are numerical descriptors that come from a compound's molecular structure. They are frequently employed in many fields of chemistry and drug discovery because they give information about the topology and connectivity of atoms within molecules [7], [8].

The Sombor indices are a group of topological indices that Milan Randić first described. They offer information about the molecular structure and characteristics and are based on a graph-theoretical approach. Boiling temperatures, refractivity, and toxicity are just a few of the physicochemical qualities of molecules that the Sombor indices have been used to predict [9], [10].

## 2 Literature review

The Sombor graph parameter was published in 2021, and it quickly attracted the scholars. On this particular index, there isn't a lot of literature. Specific graph operations and monogenic semigroup graphs are used to illustrate this Sombor index [11]. The Sombor graph parameter is utilized to analyze the Zagreb graph indices. Additionally, some extremal graph properties are evaluated in [4].

In [12], for topological influence, and particularly Sombor index is associated with molecular (atomic) orbitals. In [13], produced the sharp bounds on the Sombor graph parameters under discussion for a few generalized classes of graphs. In [14], the researchers discovered the extremal bounds of the graphs by utilizing the Sombor index. They established the some extremal class of the graphs. The author determined the Zagreb indices by utilizing the Sombor index in [15].

Additionally, certain lower and upper boundaries were provided by the Sombor index and its invariants. In [16], the Sombor index and degree-related properties are used to analyze the simplicial networks. The [17] to learn more about benzenoid hydrocarbons and their uses in the Sombor graph parameters teachnique, and also their boiling points. [18] researchers described the usage of Sombor graph parameter for chemical point of view, as well its application. Polymers produced dendrimers as stated in the quote [19], according to their analysis is critique to [20]. In [21], phenylene chains, hexagonal chains, and they measured the values of associate Sombor index. The features of Sombor index, features of molecular graphs, and their usage of other topological indices are discussed in [22], [23], [24], [25], [26], [27]. Researchers found the conjecture, Sombor graph parameter and its invariants in [28] cited there. In [29], [30], to learn more about the Sombor index and their result, read can see the cited reference. Found the latest result on Sombor graph parameter and its versions in [31].

We have discussed the basic definitions and methodology of paper work, in section 3, and derived the results of degree-based topological indices from the geometric perspective of a Y-shaped junction in section 4. In section 5, we have proved the theorem related to degree-based topological indices from the geometric perspective of the first invariant of Y-shaped junctions. In section 6, we have proved the theorem related to degree-based topological indices from the geometric perspective of the second invariant of Y-shaped junctions. Second last section, we prove the theorem related to the third variant of Y-shaped junctions. In the last section, we discussed the conclusion.

## 3 Basic formulations and methodology

Consider *G* be a simple, nontrivial, and connected graph, with vertex and edges, set V(G) and E(G), respectively. Assume that ϒ is a vertex, chosen from V(G) of a under discussion graph, the count of edges is incident to vertex *k*, then it said to a degree of taken vertex, which is under discussion graph, and symbolically written as ${ϒ}_{k}\in \Gamma (G)$ and which is different, then indicate the considered graphs is irregular, if not, then it is regular.

In Fig. 1, the origin is symbolized by *O*, and the degree points of the edge are denoted by *A*. In the Euclidean metric, *OA* is the distance between the points *O* and *A* is equal to $\sqrt{{d_{ϒ}}^{2}+{d_{\Gamma }}^{2}}$. The Sombor graph parameter is defined by adding this work on all the edges in [32] and it is expressed as in Equation (1) to (7):

(1) $$SO(G)=\sum_{ϒ\Gamma \in E(G)}dist(AO)=\sum_{ϒ\Gamma \in E(G)}\sqrt{{d_{ϒ}}^{2}+{d_{\Gamma }}^{2}}.$$

Recently, researcher used the same process to create some Sombor graph parameters. The first invariant of Sombor graph parameter was established by applying the region between the two edges and it is defined as:

(2) $$SO_{1}(G)=\sum_{ϒ\Gamma \in E(G)}area(ABO)=\sum_{ϒ\Gamma \in E(G)}\frac{1}{2}\left|{d_{ϒ}}^{2}-{d_{\Gamma }}^{2}\right|.$$

The second invariant of the Sombor graph parameter was derived with help of geometrical aspects of angle between two edges and it is defined as:

(3) $$SO_{2}(G)=\sum_{ϒ\Gamma \in E(G)}\sin\alpha =\sum_{ϒ\Gamma \in E(G)}\left|\frac{{d_{ϒ}}^{2}-{d_{\Gamma }}^{2}}{{d_{ϒ}}^{2}+{d_{\Gamma }}^{2}}\right|.$$

The third version of Sombor graph parameter was developed by applying the concept of perimeter of circumcircle on triangle ABO, and it is defined as:

(4) $$SO_{3}(G)=\sum_{ϒ\Gamma \in E(G)}perim(\Gamma_{c})=\sum_{ϒ\Gamma \in E(G)}\sqrt{2}\left(\frac{{d_{ϒ}}^{2}+{d_{\Gamma }}^{2}}{d_{ϒ}+d_{\Gamma }}\right)\pi .$$

In order to establish the fourth version of the Sombor graph parameter, the triangular ABO's circumcircle area was utilized:

(5) $$SO_{4}(G)=\sum_{ϒ\Gamma \in E(G)}area(\Gamma_{c})=\sum_{ϒ\Gamma \in E(G)}\frac{1}{2}{\left(\frac{{d_{ϒ}}^{2}+{d_{\Gamma }}^{2}}{d_{ϒ}+d_{\Gamma }}\right)}^{2}\pi .$$

The perimeter of inscribed circle, triangle ABO, was utilized to derived the Sombor graph parameter fifth invariant.

(6) $$SO_{5}(G)=\sum_{ϒ\Gamma \in E(G)}perim(\Gamma_{ϒ})=\sum_{ϒ\Gamma \in E(G)}2\pi \left(\frac{\left|{d_{ϒ}}^{2}-{d_{\Gamma }}^{2}\right|}{\sqrt{2}+2\sqrt{{d_{ϒ}}^{2}+{d_{\Gamma }}^{2}}}\right).$$

The sixth invariant of the Sombor graph parameter was produced by geometrically adjusting the size of the triangle's incircle and it is defined as:

(7) $$SO_{6}(G)=\sum_{ϒ\Gamma \in E(G)}area(\Gamma_{ϒ})=\sum_{ϒ\Gamma \in E(G)}{\left(\frac{{d_{ϒ}}^{2}-{d_{\Gamma }}^{2}}{\sqrt{2}+2\sqrt{{d_{ϒ}}^{2}+{d_{\Gamma }}^{2}}}\right)}^{2}\pi .$$

Particularly, $SO_{1},SO_{2},SO_{3},SO_{4},SO_{5},SO_{6}$ are only used to check the graph inequality, and assuming that the graph under examination is regular, these indices are equal to zero.

## 4 Degree–based topological indices from geometric perspective of Y-shaped junctions

Carbon atoms are organized in a hexagonal lattice to form the cylindrical nanotubes. They can be compared to graphene sheets that have been rolled up. Graphene is a two-dimensional form of carbon. Because of their distinctive qualities, carbon nanotubes (CNTs) are useful in a variety of fields, including electronics, nanotechnology, and materials science. These characteristics include high mechanical strength, outstanding electrical conductivity, and thermal stability.

Single-walled carbon nanotubes (SWCNTs) and multi-walled carbon nanotubes (MWCNTs) are the two primary forms of CNTs. MWCNTs are made up of numerous concentric graphene cylinders, while SWCNTs are made up of a single graphene sheet wrapped into a cylinder. The term “carbon-based nano-junctions” describes the joining or intersection of various carbon-based nanostructures, such as nanotubes. These junctions can be created using a variety of techniques, including altering and fusing separate nanotubes, adding flaws or functional groups, or developing hybrid structures using different nanomaterials.

Due to its potential use in molecular devices, energy storage, nanoelectronics, and optoelectronics, carbon-based nano-junctions have garnered a lot of attention. By adjusting the structure, composition, and connectivity at the junctions, they provide distinctive electronic and transport capabilities that may be tweaked. Scientists are now conducting research and development in this area, examining various production methods and examining the characteristics and behaviors of nano-junctions made of carbon. In order to build and create new nanoscale systems and devices, these research seek to comprehend their underlying properties.

**Figure 2:**
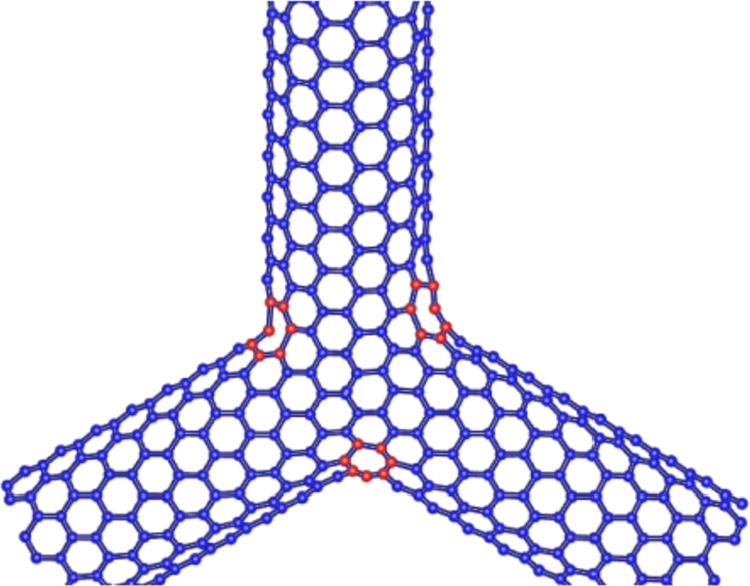
Y-shaped junction *G*_*β*_(*γ*,*γ*).

A Y-shaped junction $G_{\beta }(\gamma ,\gamma )$, where γ≥4, (even) and β≥1, is a chemical nanostructure constructed by joining three armchair carbon single-walled nanotubes and an armchair central region. The vertex degree behavior of a under consideration Y-shaped junction $G_{\beta }(\gamma ,\gamma )$ is given in Table 1, and the graph shown in the Fig. 2. For more detail on the Y-shaped junction $G_{\beta }(\gamma ,\gamma )$ and its other variant we will refer to see [33].

**Table 1:** Edge partition of Y-shaped junction *G*_*β*_(*γ*,*γ*), according to the degrees of their terminal vertex.

Edge-type	(*d*_ϒ_,*d*_Γ_)	Frequency(f)
*E*_1_	(2,2)	6*t*
*E*_2_	(2,3)	12*t*
*E*_3_	(3,3)	9*t*^2^ + 36*βt* + 3*t* + 9

Theorem 4.1 *Let* $G_{\beta }(\gamma ,\gamma )$ *be derived by using the Y-Junction of nanotubes, with the help of variational values* t≥2,γ=2t *and* β≥1 *. Then, we have*

*First invariant of Sombor graph parameter is:*

$$SO_{1}\left(G_{\beta }(\gamma ,\gamma )\right)=30t.$$

Proof First invariant of Sombor graph parameter is utilized in equation (2), and it is expressed as:

$$SO_{1}\left(G_{\beta }(\gamma ,\gamma )\right)=\sum_{ϒ\Gamma \in E(G)}area(ABO)=\sum_{ϒ\Gamma \in E(G)}\frac{1}{2}\left|{d_{ϒ}}^{2}-{d_{\Gamma }}^{2}\right|$$

To derive the above formula by applying the Geometric aspect of the topological indexes, we will utilize the degree behavior of a vertex of the network under discussion. The degree and edge type behavior of the network is listed in Table 1. We developed the desired outcome by putting the values of $\left(d_{ϒ},d_{\Gamma }\right)$, and also inserting the values in above formulation. Then, we get

$$SO_{1}\left(G_{\beta }(\gamma ,\gamma )\right)=\frac{1}{2}\left[\left|2^{2}-2^{2}\right|(6t)+\left|2^{2}-3^{2}\right|(12t)+\left|3^{2}-3^{2}\right|(9t^{2}+36\beta t+3t+9)\right]=\left(30t\right).$$

 □

Theorem 4.2 *Let* $G_{\beta }(\gamma ,\gamma )$ *be a graph, which is derived from a Y-Junction of nanotubes, with the help of variational parameter* t≥2,γ=2t *and* β≥1 *. Then*

*Second invariant of Sombor graph parameter is:*

$$SO_{2}\left(G_{\beta }(\gamma ,\gamma )\right)=(4.6154)t.$$

Proof Second invariant of Sombor graph parameter is utilized in equation (3), and it is expressed as:

$$SO_{2}\left(G_{\beta }(\gamma ,\gamma )\right)=\sum_{ϒ\Gamma \in E(G)}\sin\alpha =\sum_{ϒ\Gamma \in E(G)}\left|\frac{{d_{ϒ}}^{2}-{d_{\Gamma }}^{2}}{{d_{ϒ}}^{2}+{d_{\Gamma }}^{2}}\right|$$

To derive the above formula by applying the Geometric aspect of the topological indexes, we will utilize the degree behavior of a vertex of the network under discussion. The degree and edge type behavior of the network is listed in Table 1. We developed the desired outcome by putting the values of $\left(d_{ϒ},d_{\Gamma }\right)$, and also inserting the values in above formulation. Then, we get

$$SO_{2}\left(G_{\beta }(\gamma ,\gamma )\right)=\left[\left|\frac{2^{2}-2^{2}}{2^{2}+2^{2}}\right|(6t)+\left|\frac{2^{2}-3^{2}}{2^{2}+3^{2}}\right|(12t)+\left|\frac{3^{2}-3^{2}}{3^{2}+3^{2}}\right|(9t^{2}+36\beta t+3t+9)\right],=\left(4.6154\right)t.$$

 □

Theorem 4.3 *Let* $G_{\beta }(\gamma ,\gamma )$ *be a graph, which is derived from a Y-Junction of nanotubes, with the help of variational parameter* t≥2,γ=2t *and* β≥1 *. Then*

*Third invariant of Sombor graph parameter is:*

$$SO_{3}\left(G_{\beta }(\gamma ,\gamma )\right)=\sqrt{2}\pi \left(27t^{2}+(52.2+108\beta )t+27\right).$$

Proof Third invariant of Sombor graph parameter is utilized in equation (4), and it is expressed as:

$$SO_{3}\left(G_{\beta }(\gamma ,\gamma )\right)=\sum_{ϒ\Gamma \in E(G)}\sqrt{2}\left(\frac{{d_{ϒ}}^{2}+{d_{\Gamma }}^{2}}{d_{ϒ}+d_{\Gamma }}\right)\pi$$

This formula was established by utilizing the geometric aspect of the topological indexes, but we will use the degree behavior of the vertex of the considered network. The degree and edge type behavior of considerable network are listed in Table 1. Established the desired outcome by putting the value of $\left(d_{ϒ},d_{\Gamma }\right)$, and inserting the values in formulation. Then, we get

$$SO_{3}\left(G_{\beta }(\gamma ,\gamma )\right)=\sqrt{2}\pi \left[\frac{2^{2}+2^{2}}{2+2}(6t)+\frac{2^{2}+3^{2}}{2+3}(12t)+\frac{3^{2}+3^{2}}{3+3}(9t^{2}+36\beta t+3t+9)\right],=\sqrt{2}\pi \left(27t^{2}+(52.2+108\beta )t+27\right).$$

 □

Theorem 4.4 *Let* $G_{\beta }(\gamma ,\gamma )$ *be a graph, which is derived from a Y-Junction of nanotubes, with the help of variational parameter* t≥2,γ=2t *and* β≥1 *. Then*

*Fourth invariant of Sombor graph parameter is:*

$$SO_{4}\left(G_{\beta }(\gamma ,\gamma )\right)=\frac{1}{2}\pi \left(81t^{2}+(132.2+324\beta )t+81\right).$$

Proof Fourth invariant of Sombor graph parameter is utilized in equation (5), and it is expressed as:

$$SO_{4}\left(G_{\beta }(\gamma ,\gamma )\right)=\sum_{ϒ\Gamma \in E(G)}\frac{1}{2}{\left(\frac{{d_{ϒ}}^{2}+{d_{\Gamma }}^{2}}{d_{ϒ}+d_{\Gamma }}\right)}^{2}\pi$$

This formula was established by utilizing the geometric aspect of the topological indexes, but we will use the degree behavior of the vertex of the considered network. The degree and edge type behavior of considerable network are listed in Table 1. Established the desired outcome by putting the value of $\left(d_{ϒ},d_{\Gamma }\right)$, and inserting the values in formulation. Then, we get

$$SO_{4}\left(G_{\beta }(\gamma ,\gamma )\right)=\frac{1}{2}\pi \left[{\left(\frac{2^{2}+2^{2}}{2+2}\right)}^{2}(6t)+{\left(\frac{2^{2}+3^{2}}{2+3}\right)}^{2}(12t)+{\left(\frac{3^{2}+3^{2}}{3+3}\right)}^{2}(9t^{2}+36\beta t+3t+27)\right],=\frac{1}{2}\pi \left(81t^{2}+(132.2+324\beta )t+81\right).$$

 □

Theorem 4.5 *Let* $G_{\beta }(\gamma ,\gamma )$ *be a graph, which is derived from a Y-Junction of nanotubes, with the help of variational parameter values* t≥2,γ=2t *and* β≥1 *. Then*

*Fifth invariant of Sombor graph parameter is:*

$$SO_{5}\left(G_{\beta }(\gamma ,\gamma )\right)=2\pi \left(13.9125\right)t.$$

Proof Fifth invariant of Sombor graph parameter is utilized in equation (6), and it is expressed as:

$$SO_{5}\left(G_{\beta }(\gamma ,\gamma )\right)=\sum_{ϒ\Gamma \in E(G)}2\pi \left(\frac{\left|{d_{ϒ}}^{2}-{d_{\Gamma }}^{2}\right|}{\sqrt{2}+2\sqrt{{d_{ϒ}}^{2}+{d_{\Gamma }}^{2}}}\right).$$

This formula was established by utilizing the geometric aspect of the topological indexes, but we will use the degree behavior of the vertex of the considered network. The degree and edge type behavior of considerable network are listed in Table 1. Established the desired outcome by putting the value of $\left(d_{ϒ},d_{\Gamma }\right)$, and inserting the values in formulation. Then, we get

$$SO_{5}\left(G_{\beta }(\gamma ,\gamma )\right)=2\pi \left[\frac{\left|4-4\right|}{\sqrt{2}+2\sqrt{4+4}}(6t)+\frac{\left|4-9\right|}{\sqrt{2}+2\sqrt{4+9}}(12t\right)+\frac{\left|9-9\right|}{\sqrt{2}+2\sqrt{9-9}}(9t^{2}+36\beta t+3t+27)],=2\pi \left(13.9125\right)t.$$

 □

Theorem 4.6 *Let* $G_{\beta }(\gamma ,\gamma )$ *be a graph, which is derived from a Y-Junction of nanotubes, with the help of variational parameter values* t≥2,γ=2t *and* β≥1 *. Then*

*Sixth invariant of Sombor graph parameter is:*

$$SO_{6}\left(G_{\beta }(\gamma ,\gamma )\right)=\pi \left(4.0325\right)t.$$

Proof Sixth invariant of Sombor graph parameter is utilized in equation (7), and it is expressed as:

$$SO_{6}\left(G_{\beta }(\gamma ,\gamma )\right)=\sum_{ϒ\Gamma \in E(G)}{\left(\frac{{d_{ϒ}}^{2}-{d_{\Gamma }}^{2}}{\sqrt{2}+2\sqrt{{d_{ϒ}}^{2}+{d_{\Gamma }}^{2}}}\right)}^{2}\pi .$$

This formula was established by utilizing the geometric aspect of the topological indexes, but we will use the degree behavior of the vertex of the considered network. The degree and edge type behavior of considerable network are listed in Table 1. Established the desired outcome by putting the value of $\left(d_{ϒ},d_{\Gamma }\right)$, and inserting the values in formulation. Then, we get

$$SO_{6}\left(G_{\beta }(\gamma ,\gamma )\right)=\pi \left[{\left(\frac{4-4}{\sqrt{2}+2\sqrt{4+4}}\right)}^{2}(6t)+{\left(\frac{4-9}{\sqrt{2}+2\sqrt{4+9}}\right)}^{2}(12t\right)+{\left(\frac{9-9}{\sqrt{2}+2\sqrt{9+9}}\right)}^{2}(9t^{2}+36\beta t+3t+27)],=\pi \left(4.0325\right)t.$$

 □

## 5 Degree–based topological indices from geometric perspective of first variant of Y-shaped junctions

A first variant of Y-shaped junction $G_{\beta }^{1}(\gamma ,\gamma )$, where γ≥4, (even) and β≥1, is a chemical nanostructure constructed by joining three armchair carbon single-walled nanotubes and an armchair central region but one nanotube has 4*γ* degree one vertices. The vertex degree behavior of the considered Y-shaped junction $G_{\beta }^{2}(\gamma ,\gamma )$ is given in Table 2.

**Table 2:** Edge partition of first variant of Y-shaped junction $G_{\beta }^{1}(\gamma ,\gamma )$, according to the degrees of their terminal vertex.

Edge-type	(*d*_ϒ_,*d*_Γ_)	Frequency
*E*_1_	(1,3)	4*t*
*E*_2_	(2,2)	4*t*
*E*_3_	(2,3)	8*t*
*E*_4_	(3,3)	9*t*^2^ + 36*βt* + 9*t* + 9

In a similar, by applying the definitions are provided in the Equations (2) to (7) and calculating the values of edge division are listed in the Table 2, the following theorems can be computed for the first variant of Y-shaped junction $G_{\beta }^{1}(\gamma ,\gamma )$.

Theorem 5.1 *Let*$G_{\beta }^{1}(\gamma ,\gamma )$*be a graph constructed from first variant of Y-Junction of nanotubes, with parametric values*t≥2,γ=2t*and*β≥1*. Then*

*First invariant of Sombor graph parameter is:*

$$SO_{1}\left(G_{\beta }^{1}(\gamma ,\gamma )\right)=\left(36\right)t.$$

Theorem 5.2 *Let*$G_{\beta }^{1}(\gamma ,\gamma )$*be a graph, which is derived from first variant of Y-Junction of nanotubes, with the help of variational parameter*t≥2,γ=2t*and*β≥1*. Then*

*Second invariant of Sombor graph parameter is:*

$$SO_{2}\left(G_{\beta }^{1}(\gamma ,\gamma )\right)=\left(6.2769\right)t.$$

Theorem 5.3 *Let*$G_{\beta }^{1}(\gamma ,\gamma )$*be a graph, which is derived from first variant of Y-Junction of nanotubes, with the help of variational parameter*t≥2,γ=2t*and*β≥1*. Then*

*Second invariant of Sombor graph parameter is:*

$$SO_{3}\left(G_{\beta }^{1}(\gamma ,\gamma )\right)=\sqrt{2}\left(27+(108\beta +65.8)t+27t^{2}\right)\pi .$$

Theorem 5.4 *Let*$G_{\beta }^{1}(\gamma ,\gamma )$*be a graph, which is derived from first variant of Y-Junction of nanotubes, with the help of variational parameter*t≥2,γ=2t*and*β≥1*. Then*

*Third invariant of Sombor graph parameter is:*

$$SO_{4}\left(G_{\beta }^{1}(\gamma ,\gamma )\right)=\frac{1}{2}\pi \left(81t^{2}+(176.08+324\beta t+81)\right).$$

Theorem 5.5 *Let*$G_{\beta }^{1}(\gamma ,\gamma )$*be a graph, which is derived from first variant of Y-Junction of nanotubes, with the help of variational parameter*t≥2,γ=2t*and*β≥1*. Then*

*Fifth invariant of Sombor graph parameter is:*

$$SO_{5}\left(G_{\beta }^{1}(\gamma ,\gamma )\right)=2\pi \left(17.6\right)t.$$

Theorem 5.6 *Let*$G_{\beta }^{1}(\gamma ,\gamma )$*be a graph, which is derived from first variant of Y-Junction of nanotubes, with the help of variational parameters*t≥2,γ=2t*and*β≥1*. Then*

*Sixth invariant of Sombor graph parameter is:*

$$SO_{6}\left(G_{\beta }^{1}(\gamma ,\gamma )\right)=\pi \left(6.9629\right)t.$$

## 6 Degree–based topological indices geometric perspective of second variant of Y-shaped junctions

A second variant of Y-shaped junction $G_{\beta }^{2}(\gamma ,\gamma )$, where γ≥4, (even) and β≥1, is a chemical nanostructure constructed by joining three armchair carbon single-walled nanotubes and an armchair central region but two nanotube has 8*γ* degree one vertices. The vertex degree behavior of considered Y-shaped junction $G_{\beta }^{2}(\gamma ,\gamma )$ is given in Table 3.

**Table 3:** Edge partition of second variant of Y-shaped junction $G_{\beta }^{2}(\gamma ,\gamma )$, according to the degrees of their terminal vertex.

Edge-type	(*d*_ϒ_,*d*_Γ_)	Frequency
*E*_1_	(1,3)	8*t*
*E*_2_	(2,2)	2*t*
*E*_3_	(2,3)	4*t*
*E*_4_	(3,3)	9*t*^2^ + 36*βt* + 15*t* + 9

In a similar, by applying the definition are provided in the Equations (2) to (7) and calculating the values of edge division are listed in the Table 3, the following theorems can be computed for the second variant of Y-shaped junction $G_{\beta }^{2}(\gamma ,\gamma )$.

Theorem 6.1 *Let* $G_{\beta }^{2}(\gamma ,\gamma )$ *be a graph constructed from second variant of Y-Junction of nanotubes, with parametric values* t≥2,γ=2t *and* β≥1 *. Then*

*First invariant of Sombor graph parameter is:*

$$SO_{1}\left(G_{\beta }^{2}(\gamma ,\gamma )\right)=\left(42\right)t.$$

Theorem 6.2 *Let* $G_{\beta }^{2}(\gamma ,\gamma )$ *be a graph constructed from second variant of Y-Junction of nanotubes, with parametric values* t≥2,γ=2t *and* β≥1 *. Then*

*Second invariant of Sombor graph parameter is:*

$$SO_{2}\left(G_{\beta }^{2}(\gamma ,\gamma )\right)=\left(7.9385\right)t.$$

Theorem 6.3 *Let* $G_{\beta }^{2}(\gamma ,\gamma )$ *be a graph constructed from second variant of Y-Junction of nanotubes, with parametric values* t≥2,γ=2t *and* β≥1 *. Then*

*Third invariant of Sombor graph parameter is:*

$$SO_{3}\left(G_{\beta }^{2}(\gamma ,\gamma )\right)=\sqrt{2}\pi \left(27t^{2}+(79.4+108\beta )t+27\right).$$

Theorem 6.4 *Let* $G_{\beta }^{2}(\gamma ,\gamma )$ *be a graph constructed from second variant of Y-Junction of nanotubes, with parametric values* t≥2,γ=2t *and* β≥1 *. Then*

*Fourth invariant of Sombor graph parameter is:*

$$SO_{4}\left(G_{\beta }^{2}(\gamma ,\gamma )\right)=\frac{1}{2}\pi \left(81t^{2}+(135.2+324\beta )t+81\right).$$

Theorem 6.5 *Let* $G_{\beta }^{2}(\gamma ,\gamma )$ *be a graph constructed from second variant of Y-Junction of nanotubes, with parametric values* t≥2,γ=2t *and* β≥1 *. Then*

*Fifth invariant of Sombor graph parameter is:*

$$SO_{5}\left(G_{\beta }^{2}(\gamma ,\gamma )\right)=2\pi \left(21.1776\right)t.$$

Theorem 6.6 *Let* $G_{\beta }^{2}(\gamma ,\gamma )$ *be a graph constructed from second variant of Y-Junction of nanotubes, with parametric values* t≥2,γ=2t *and* β≥1 *. Then*

*Sixth invariant of Sombor graph parameter is:*

$$SO_{6}\left(G_{\beta }^{2}(\gamma ,\gamma )\right)=\pi \left(9.8934\right)t.$$

## 7 Degree–based topological from geometric perspective indices of third variant of Y-shaped junctions

A third variant of Y-shaped junction $G_{\beta }^{3}(\gamma ,\gamma )$, where γ≥4, (even) and β≥1, is a chemical nanostructure constructed by joining three armchair carbon single-walled nanotubes and an armchair central region but three nanotube has 12*γ* degree one vertices. The vertex degree behavior of the considered Y-shaped junction $G_{\beta }^{3}(\gamma ,\gamma )$ is given in Table 4. In a similar, by applying the definition are provided in the Equations (2) to (7) and calculating the values of edge division are listed in the Table 4, the following theorems can be computed for the third variant of Y-shaped junction $G_{\beta }^{3}(\gamma ,\gamma )$.

**Table 4:** Edge partition of third variant of Y-shaped junction $G_{\beta }^{3}(\gamma ,\gamma )$, according to the degrees of their terminal vertex.

Edge-type	(*d*_ϒ_,*d*_Γ_)	Frequency
*E*_1_	(1,3)	8*t*
*E*_2_	(3,3)	9*t*^2^ + 36*βt* + 15*t* + 9

Theorem 7.1 *Let* $G_{\beta }^{3}(\gamma ,\gamma )$ *be a graph constructed from third variant of Y-Junction of nanotubes, with parametric values* t≥2,γ=2t *and* β≥1 *. Then*

*First invariant of Sombor graph parameter is:*

$$SO_{1}\left(G_{\beta }^{3}(\gamma ,\gamma )\right)=\left(48\right)t.$$

Theorem 7.2 *Let* $G_{\beta }^{3}(\gamma ,\gamma )$ *be a graph constructed from third variant of Y-Junction of nanotubes, with parametric values* t≥2,γ=2t *and* β≥1 *. Then*

*Second invariant of Sombor graph parameter is:*

$$SO_{2}\left(G_{\beta }^{3}(\gamma ,\gamma )\right)=\left(9.6\right)t.$$

Theorem 7.3 *Let* $G_{\beta }^{3}(\gamma ,\gamma )$ *be a graph constructed from third variant of Y-Junction of nanotubes, with parametric values* t≥2,γ=2t *and* β≥1 *. Then*

*Third invariant of Sombor graph parameter is:*

$$SO_{3}\left(G_{\beta }^{3}(\gamma ,\gamma )\right)=\sqrt{2}\pi \left(27t^{2}+(93+108\beta )t+27\right).$$

Theorem 7.4 *Let* $G_{\beta }^{3}(\gamma ,\gamma )$ *be a graph constructed from third variant of Y-Junction of nanotubes, with parametric values* t≥2,γ=2t *and* β≥1 *. Then*

*Fourth invariant of Sombor graph parameter is:*

$$SO_{4}\left(G_{\beta }^{3}(\gamma ,\gamma )\right)=\frac{1}{2}\pi \left(81t^{2}+(264+324\beta )t+81\right).$$

Theorem 7.5 *Let* $G_{\beta }^{3}(\gamma ,\gamma )$ *be a graph constructed from third variant of Y-Junction of nanotubes, with parametric values* t≥2,γ=2t *and* β≥1 *. Then*

*Fifth invariant of Sombor graph parameter is:*

$$SO_{5}\left(G_{\beta }^{3}(\gamma ,\gamma )\right)=2\left(24.8101\right)\pi t.$$

Theorem 7.6 *Let* $G_{\beta }^{3}(\gamma ,\gamma )$ *be a graph constructed from third variant of Y-Junction of nanotubes, with parametric values* t≥2,γ=2t *and* β≥1 *. Then*

*Sixth invariant of Sombor graph parameter is:*

$$SO_{6}\left(G_{\beta }^{3}(\gamma ,\gamma )\right)=\left(12.8238\right)\pi t.$$

## 8 Conclusion

Only Sombor graph parameter has been studied from geometric prospective, among the vertex-degree- based indexes. The first invariant of Sombor graph parameter is area based, which is proposed by Euclidean geometry. Sixth, fourth, and second invariant of Sombor graph parameter were established by via angular orientation. We worked on recently developed Sombor graph parameter for various nanotube Y-junctions. Y-junction the basic one then its three further variants are considered. Particularly, labeled as $G_{\beta }(\gamma ,\gamma )$, $G_{\beta }^{1}(\gamma ,\gamma )$, $G_{\beta }^{2}(\gamma ,\gamma )$, and $G_{\beta }^{3}(\gamma ,\gamma )$.

### 8.1 Future direction

In future, one can consider energy related graph theoretical parameters to study these nanostructures, like $G_{\beta }(\gamma ,\gamma )$, $G_{\beta }^{1}(\gamma ,\gamma )$, $G_{\beta }^{2}(\gamma ,\gamma )$, and $G_{\beta }^{3}(\gamma ,\gamma )$. There are many energy and eigen value related topological indices available in the literature and those can be researched for future work.

## CRediT authorship contribution statement

M. Imran, R. Ismail, M. Azeem, M. K. Jamil, and E. H. A. Al-Sabri: conceived and designed the experiments; performed the experiments; analyzed and interpreted the data; contributed reagents, materials, analysis tools or data; wrote the paper.

## Declaration of Competing Interest

The authors declare no conflict of interest.

## Data Availability

Data included in article/supplementary material/referenced in article.
